# Intentional inhibition but not source memory is related to hallucination-proneness and intrusive thoughts in a university sample

**DOI:** 10.1016/j.cortex.2018.12.020

**Published:** 2019-04

**Authors:** Ben Alderson-Day, David Smailes, Jamie Moffatt, Kaja Mitrenga, Peter Moseley, Charles Fernyhough

**Affiliations:** a Department of Psychology, Durham University, Durham, UK; b Department of Psychology, University of Northumbria, Newcastle-Upon-Tyne, UK; c School of Psychology, University of Sussex, Falmer, UK; d Department of Psychology, University of Central Lancashire, Preston, UK

**Keywords:** Hallucinations, Psychosis, Source monitoring, Working memory, Cognitive control, Executive function

## Abstract

Proneness to unusual perceptual states – such as auditory or visual hallucinations – has been proposed to exist on a continuum in the general population, but whether there is a cognitive basis for such a continuum remains unclear. Intentional cognitive inhibition (the ability to wilfully control thoughts and memories) is one mechanism that has been linked to auditory hallucination susceptibility, but most evidence to date has been drawn from clinical samples only. Moreover, such a link has yet to be demonstrated over and above relations to other cognitive skills (source monitoring) and cognitive states (intrusive thoughts) that often correlate with both inhibition and hallucinations. The present study deployed two tests of intentional inhibition ability – the Inhibition of Currently Irrelevant Memories (ICIM) task and Directed Forgetting (DF) task – and one test of source monitoring (a source memory task) to examine how cognitive task performance relates to self-reported i) auditory hallucination-proneness and ii) susceptibility to intrusive thoughts in a non-clinical student sample (*N* = 76). Hierarchical regression analyses were used to assess the independent and combined contributions of task performance to proneness scores. ICIM performance but not DF or source memory scores were significantly related to both hallucination-proneness and intrusive thoughts. Further analysis suggested that intrusive thoughts may mediate the link between intentional inhibition skills and auditory hallucination-proneness, suggesting a potential pathway from inhibition to perception via intrusions in cognition. The implications for studying cognitive mechanisms of hallucination and their role in “continuum” views of psychosis-like experiences are discussed.

## 1 Introduction

Psychosis and psychosis-like experiences have been proposed to occur on a continuum linking clinical and non-clinical populations (Johns and van Os, 2001, Strauss, 1969). Accordingly, the cognitive mechanisms that may underlie unusual beliefs (delusions) and perceptions (hallucinations) have also been posited to exist on a continuum, with variations in cognitive skills acting as an extended phenotype of psychosis susceptibility (Allen et al., 2006, Freeman et al., 2008, Kelleher et al., 2013). While the notion of a psychosis continuum has been challenged (David, 2010), evidence of cognitive processes being atypical in similar ways for both clinical and non-clinical hallucination-prone groups has been treated as a key piece of supporting evidence for a continuum view (Brookwell, Bentall, & Varese, 2013). In the case of auditory hallucinations (AH), one putative underlying mechanism is cognitive inhibition (Badcock and Hugdahl, 2012, Waters et al., 2012).

AH are typically intrusive and uncontrollable experiences (David, 1999; although see; Powers, Kelley, & Corlett, 2017), making problems with inhibitory control a plausible part of their causal explanation. General difficulties with inhibition are evident in people with schizophrenia (Westerhausen, Kompus, & Hugdahl, 2011), 60–90% of whom experience AH (Bauer et al., 2011). However, specific relations between inhibitory control and hallucinations are relatively scarce in the literature. Instead, it has been argued that a specific problem with “intentional cognitive inhibition” – the ability to consciously and willingly suppress information from working memory – plays an important role in AH (Badcock, Waters, Maybery, & Michie, 2005). Evidence for this has largely come from continuous recognition paradigms such as the Inhibition of Current Irrelevant Memories (ICIM) task (Schnider and Ptak, 1999, Waters et al., 2006), in which participants must learn to recognise a series of picture targets, then ignore the impulse to respond to them on subsequent rounds containing new targets. Participants with schizophrenia and AH make more false alarms on the ICIM than patients without AH and healthy controls, and this correlates with AH severity (Badcock et al., 2005, Waters et al., 2003). Supporting evidence has also come from studies of “directed forgetting”, in which participants are instructed to forget previously learned words or word lists, but then later tested on their recall (Bjork and Woodward, 1973, Geiselman et al., 1983). While healthy participants typically show a directed forgetting effect (i.e., reduced recall for words in “forget” versus “remember” lists; Conway, Harries, Noyes, Racsmany, & Frankish, 2000), participants with schizophrenia forget fewer words (Racsmány et al., 2008) and this correlates with hallucination severity in patients with AH (Soriano, Jiménez, Román, & Bajo, 2009).

Findings such as these have been used to argue for inhibition playing an important role in understanding hallucinations more generally, both clinically (across various modalities and diagnoses), and as a marker for hallucination susceptibility in the general population (Badcock and Hugdahl, 2014, Ford et al., 2014, Jardri et al., 2016). However, evidence is more limited for intentional inhibition specifically being linked to hallucination-proneness in non-clinical populations (e.g., hearing a telephone ring when it has not). In one study, Paulik, Badcock, and Maybery (2007) found that those high in hallucination-proneness were more likely to make false alarms on the ICIM task than those low in hallucination-proneness. This was partially replicated by Badcock, Mahfouda, and Maybery (2015), who observed poorer ICIM performance in healthy individuals high in hypomanic traits and hallucination-proneness. There is also evidence of hallucination-proneness in non-clinical samples being related to false alarms during free recall (Brébion, Larøi, & Van der Linden, 2010) and errors on false memory tasks (Sugimori, Asai, & Tanno, 2011). However, the extent to which unwitting errors during general recall represent *intentional* inhibition (in the same way that ICIM and directed forgetting tasks are proposed to index) is unclear*,* given that such errors are usually taken to reflect unintentional and unconscious inhibitory processes (Nigg, 2000, Paulik et al., 2008). Beyond this, systematic investigation of the relations between intentional inhibition and hallucination-proneness in healthy samples has not been examined, either on the ICIM, or on alternative tasks such as directed forgetting.^1^

One concern about the link between AH and intentional inhibition is specificity, given that other cognitive and psychopathological factors could plausibly mediate any relationship between the two. First, hallucination-proneness has often been linked to atypical source monitoring (Brookwell et al., 2013) i.e., the ability to track and distinguish the origin of information (Johnson, Hashtroudi, & Lindsay, 1993). Disruptions to source monitoring have long been proposed as the process by which internal cognitions could be experienced as coming from another agent, via disruption to internal predictive models (Frith, 1992) and biases to attribute sensations to external sources (Bentall, 1990). Clinical participants with frequent AH often have difficulties with source monitoring (Brébion et al., 2012, Moritz et al., 2003, Woodward et al., 2007) with more mixed evidence reported in non-clinical samples (Garrison et al., 2017, Larøi et al., 2004). As noted by Badcock et al. (2005), there is a source monitoring demand on the ICIM, given that participants must track targets from current and previous rounds in order to respond correctly. It has also been suggested that the directed forgetting effect is driven by demands of monitoring the change in context between the “forget” and “remember” lists, rather than intentional suppression of the “forget” list (Sahakyan et al., 2013, Sahakyan and Kelley, 2002). As such, relations between inhibition and hallucination-proneness may therefore actually reflect indirect demands on source monitoring, rather than a direct or independent pathway for hallucinatory experience. This needs to be tested empirically by including measures of both inhibition and source monitoring in the same study.

Second, intentional inhibition performance has been studied in relation to a range of conditions characterised not by AH, but by intrusive thoughts (such as obsessive-compulsive disorder; Badcock et al., 2007, Bannon et al., 2002). The strong overlap between intrusive thoughts and AH (Jones and Fernyhough, 2009, Morrison et al., 1995) serves to complicate attempts to link hallucinations to inhibition. It is possible that atypical performance on tasks such as the ICIM or directed forgetting are associated with a general proneness to intrusive cognitions (e.g., Verwoerd, Wessel, & de Jong, 2009), rather than hallucination-proneness specifically. That is, a susceptibility to intrusions in cognition could be an important mediating state between inhibitory control and hallucinations, such that some thoughts then go on to be the contents of hallucinatory experiences (Morrison et al., 1995).

To address these issues, we present an investigation of intentional inhibition, source monitoring, hallucination-proneness and intrusive thoughts in a sample of non-clinical participants. We first attempted a replication of Paulik et al. (2007) by testing whether false alarms on the ICIM predicted hallucination-proneness scores on a commonly-used measure, the Launay-Slade Hallucination Scale (hypothesis 1). We then sought to extend Paulik's finding using a directed forgetting task, hypothesising that those with greater hallucination-proneness would be less effective at forgetting items when instructed to, i.e., a reduced directed forgetting effect (hypothesis 2). Using a source memory task, we examined whether source monitoring could account for any relations observed between intentional inhibition and hallucination-proneness (hypothesis 3). Finally, we also collected scores for intrusive thoughts from the White Bear Suppression Inventory (Wegner & Zanakos, 1994) to test whether performance on the above tasks primarily predicted intrusions rather than hallucination-proneness (hypothesis 4). If so, this would suggest a mediating role for intrusive cognitions between inhibition and hallucinations.

## 2 Method

### 2.1 Participants

76 participants (65 female), aged 18–28 years (M = 20.21, SD = 1.67) were recruited from university settings. Participants were required to be over 18, native English speakers, with normal or corrected-to-normal hearing or vision and no prior neurological diagnosis (these criteria were clearly stated in the study advertising and participant information prior to consent being taken). The majority of participants were white British (71.05%). The study was advertised via a departmental online participant pool, an email circular to university staff and students, social media, and word of mouth. All procedures were approved by a university research ethics committee. Participants received course credit or gift vouchers for their participation.

### 2.2 Measures

#### 2.2.1 Inhibition of Current Irrelevant Memories task

The ICIM – adapted from Paulik et al. (2007) – consisted of three runs, each containing sequential presentation of black and white line drawings (Snodgrass & Vanderwart, 1980). For each picture, participants were required to decide whether it was previously presented within the current run (a ‘target’ item). Images were displayed in the centre of a computer screen for 2000 ms with an inter-stimulus interval of 700 ms. There was a 30s break between runs one and two, and a 5-min break between runs two and three (during which time participants began an arithmetic distractor task). With each image display, participants were asked whether they had seen it before, responding with a button press (answering ‘1’ if they thought it was the first time they saw the picture, and ‘2’ if they thought it was a repeat). The order of pictures (and status as targets) changed between runs, with no targets repeating across runs. Each run included the same 60 unique images: 40 pictures were presented only once, 5 pictures were presented twice and 15 pictures were presented three times (totalling 95 image presentations). In total there were therefore 35 opportunities to identify a repeat (classed as a “hit”) and 60 opportunities per run to make a false alarm by classifying a first presentation as a repeat. However, the 20 targets from run 1 were expected to be particularly likely to prompt false alarms in run 2, while the targets from runs 1 and 2 in turn had to be resisted on run 3. Although inhibitory demands may be expected to keep increasing with each run (given the growing number of items that were previous targets), in practice false alarm rates do not change substantially from run 2 onwards (e.g., Paulik et al., 2007). We therefore followed other prior studies in using the combined number of false alarms made on runs 2 and 3 as the primary outcome on the task. Performance after run 1 is often aggregated to study intentional inhibition effects, as the observed effect (and relation to hallucinations) is thought to be generally evident from run 2 onwards (Waters et al., 2003).

#### 2.2.2 Directed forgetting task

The Directed Forgetting (DF) task was a modified version of the task used by Conway et al. (2000), which included a *forget* and a *remember* condition. In both conditions, participants viewed two lists of 10 words and were tested on their recall following a 5-min delay. Each word was presented in the middle of the computer screen for 2 s, with a 2s inter-stimulus interval. In the *forget* condition, following presentation of List 1 participants were told that what they had seen *was in fact a practice list to familiarise you with the presentation rate and type of words. You should now put these words out of mind, try to forget them and not let them interfere with learning the experimental list which will be presented now.* They then proceeded to view the words for List 2. In the *remember* condition, participants instead saw the following instructions between Lists 1 and 2: *That is the end of the words on list one. You must try to keep those in mind as you learn the second list which will be presented now*. Participants then completed a distractor task (arithmetic puzzles) for 5 min, before being asked to write down as many words as they could remember from both lists, starting with the first list (to counteract the potential interference effects of recalling list 2 items first). All participants completed both *remember* and *forget* conditions, with the order being counterbalanced across the sample. The DF effect is typically measured in terms of reduced recall on list 1 of the *forget* condition, either in comparison to list 1 for the *remember* condition (Conway et al., 2000) or list 2 of the *forget* condition (Soriano et al., 2009). These are sometimes referred to as the costs and benefits respectively of directed forgetting (Sahakyan et al., 2013).

#### 2.2.3 Source memory task (SMT)

The SMT was adapted from the version used in Experiment 2 of Garrison et al. (2017). The task comprised of a learning and a test phase. The learning phase involved presentation of 48 partially completed word phrases (e.g., bacon and e_ _). When the word ‘Other’ was displayed in the trial, participants were required to listen to the word being read out by a male voice. When the word ‘You’ was displayed, participants were instructed to complete and read the word pairs out loud. The generation and test phase were separated by a 20-min break, during which the participant completed the questionnaire pack, and a signal detection task (not reported here). During the test phase, the word pairs from the generation phase were presented again in separate trials, as well as 24 additional distractor word pairs. For each trial, participants were required to decide whether they had heard the word, spoken the word themselves, or whether they thought it was a completely new (distractor) word. The primary outcome variable was accuracy in recalling the correct source of the old items (i.e., self or other), expressed as a percentage of all items that were correctly identified as old rather than new. We also calculated an index of old-new discrimination: the proportion of trials in which old versus new trials were correctly identified (even if the self/other source was confused). Higher accuracy scores indicated better performance on the task and source monitoring abilities (Garrison et al., 2017).

#### 2.2.4 Revised Launay-Slade Hallucination Scale – auditory

This scale includes nine items derived from McCarthy-Jones and Fernyhough (2011), which itself is a version adapted from Morrison, Wells, and Nothard (2000)'s Revised LSHS. The scale consists of five statements relating to auditory hallucinations (e.g., “I hear people call my name and find that nobody has done so.”) and four statements related to visual modality of hallucinations (Items 6–9; e.g., “I see shadows and shapes when there is nothing there”). Ratings are made on a four-point Likert scale ranging from “Never” (1) to “Almost always” (4). To specifically examine auditory hallucination-proneness (as per prior studies, e.g., Waters et al., 2003), only the auditory items were included in the present analysis; visual items were collected to test for specificity only (see Supplementary Materials). Scores on the auditory subscale (LSHS-A henceforth) can range from 5 to 20, where higher scores indicate greater hallucination-proneness. The LSHS has been shown to have acceptable internal reliability (McCarthy-Jones & Fernyhough, 2011).

#### 2.2.5 White Bear Suppression Inventory

This 15-item self-report questionnaire includes five statements relating to thought intrusion and 10 items relating to thought suppression (Höping and de Jong-Meyer, 2003, Wegner and Zanakos, 1994). Ratings are made on a five-point scale ranging from “Strongly disagree” (1) to “Strongly agree” (5). To specifically examine the role of intrusive thoughts, only the 5-item intrusion subscale described by Jones and Fernyhough (2006) was used in the present analysis (WBSI-I). Scores on this subscale could range from 5 to 25, where higher scores indicate higher levels of intrusive thoughts. Previous studies have reported high levels of internal reliability for this sub-scale (e.g., Jones, Fernyhough, & Meads, 2009).

### 2.3 Procedure

All testing was carried out in a quiet laboratory room. Participants were told that they would be taking part in a study of “cognitive performance, intrusive thoughts, and unusual experiences”. Presentation of each task was with experimental software EPrime 2.0. The auditory stimuli presented in the SMT were played through over-ear Sennheiser HD206 headphones at a comfortable volume. The volume of stimuli could be adjusted during a practice trial, but no participant required this during testing. Each experimental session commenced with either the *forget* or *remember* of the DF task, depending on the order assigned to a participant. This was followed by the learning phase of the SMT, completion of the questionnaire pack, SMT test phase, ICIM, and then the remaining condition of the DF task. Both the SMT and ICIM were preceded by a short series of practice trials.

### 2.4 Data analysis

Unless otherwise specified, all data analysis was conducted in jamovi v0.9.2.9. Before hypothesis-testing, all main outcome variables were assessed for normality. Based on a combination of normality tests, QQ plot inspection and scores for skew and kurtosis, the following variables were transformed using a natural logarithm to facilitate homogeneity of residuals for regression analysis: Launay-Slade Hallucination Scale – Auditory (LSHS-A) and Visual (LSHS-V), and ICIM false alarm rates (runs 2–3). For descriptive statistics and pairwise correlations, non-transformed scores are included here for ease of interpretation. ICIM and DF task performance was analysed first using repeated measures ANOVA and paired *t*-tests to establish the presence of typical within-subjects effects on each task (i.e., an increase in false alarms from run 1 to run 2 and 3 in the former, and a difference between remember and forget conditions in the latter). Relations between inhibition performance and hallucination-proneness were then assessed using hierarchical linear regression, with LSHS-A scores as the dependent variable. The same analysis was then repeated for intrusive thoughts, with WBSI-I scores as the dependent variable. Correlations reported are Spearman's Rho correlation co-efficients.

## 3 Results

### 3.1 Descriptive statistics and within-subjects effects

**Fig. 1:**
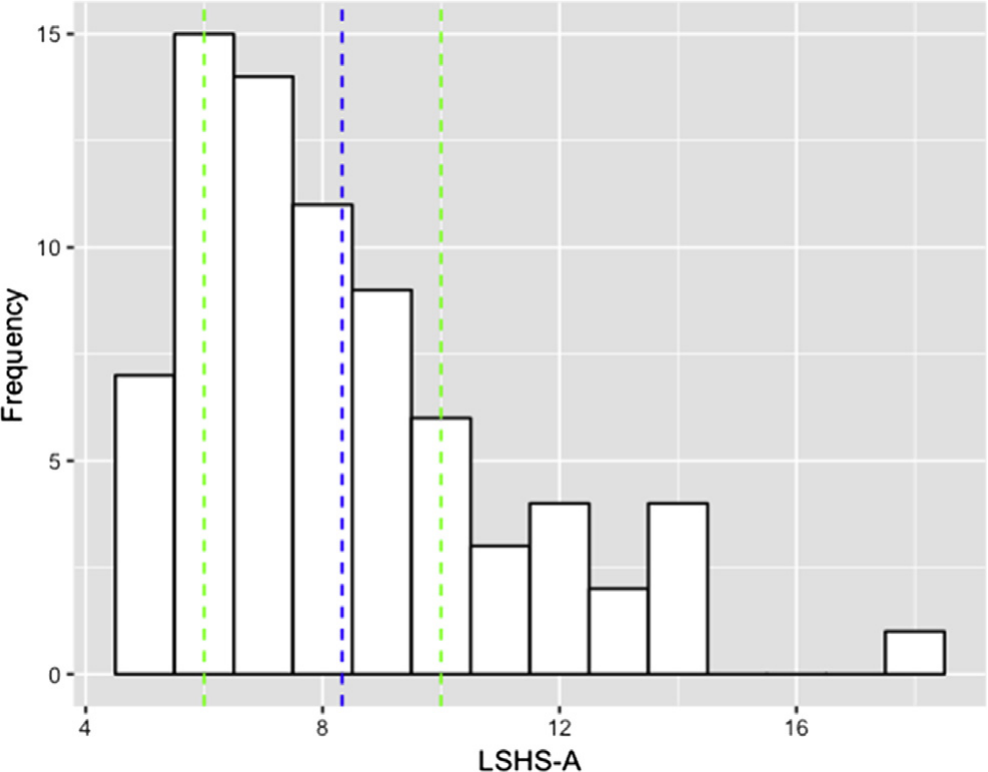
**Distribution of auditory hallucination-proneness scores**. LSHS-A = Launay-Slade Hallucination Scale – Auditory. Blue line = mean. Green lines = 25/75% quartiles. Max. score = 20.

Fig. 1 displays the distribution of hallucination-proneness scores (LSHS-A), while Table 1 displays mean scores and primary outcomes for the three tasks. As can be seen from Fig. 1, the LSHS-A distribution was positively skewed with the majority of participants reporting relatively low scores; however, this still included a quarter of participants scoring at 50% or above of the maximum score (20).

**Table 1:** Mean scores for ICIM, directed forgetting, and source memory.

	*M(SD)*	
**ICIM**	*Hits*	*False Alarms*
*Run 1*	33.28 (1.69)	1.96 (2.06)
*Run 2*	30.38 (3.75)	6.87 (5.97)
*Run 3*	31.86 (3.20)	5.51 (5.58)
**Directed Forgetting**	*Forget*	*Remember*
*Forget Condition First*		
*List 1*	5.36 (2.54)	4.90 (2.85)
*List 2*	5.95 (2.25)	5.18 (3.28)
*Remember Condition First*		
*List 1*	4.27 (3.03)	5.49 (2.78)
*List 2*	5.16 (3.18)	5.35 (2.88)
**Source Memory**	*Mean*	% Score*
*Self & Other Correct*	37.86 (4.98)	89.25 (7.13)
*Old & New Correct*	64.24 (3.82)	89.22 (5.30)

*N* = 76. ICIM = Inhibition of Current Irrelevant Memories. * For Self-Other scores, % score is out of all trials correctly classed as old (i.e., does not include self/other trials mistaken for new trials); Old/New correct is out of all trials (72).

**Fig. 2:**
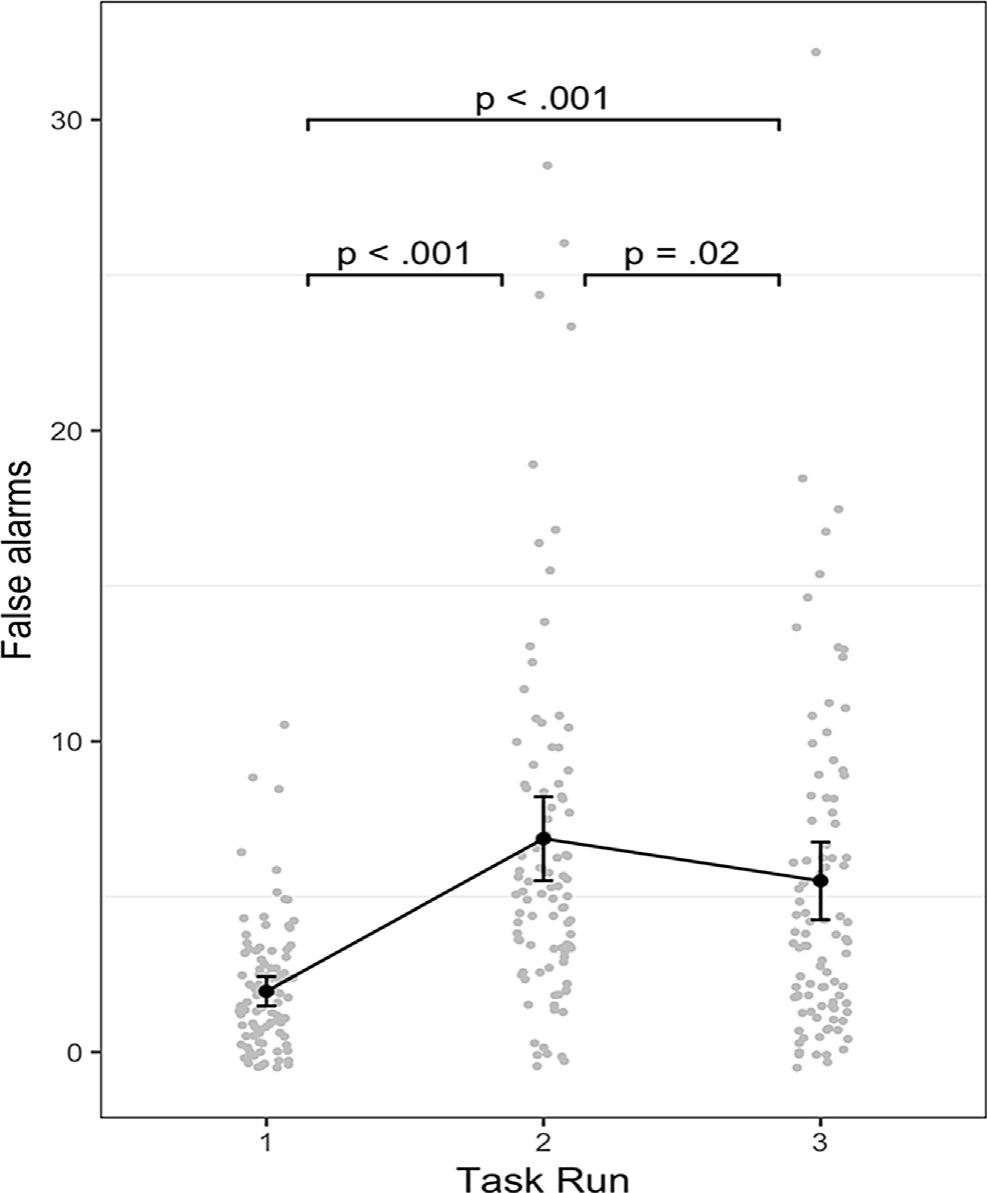
**Line graph showing number of false alarms in each run of the ICIM task**. Participants made significantly more false alarms on runs 2 and 3 of the task, compared to run 1, reflecting failure to inhibit previously seen images. Error bars = 95% confidence intervals. Dots = individual data points.

False alarms on each run of the ICIM were analysed using repeated measures ANOVA, indicating a significant effect of run, *F*(2, 150) = 37.39, *p* < .001, μ^2^_p_ = .33. As Fig. 2 shows, participants made significantly more false alarms in run 2 compared to run 1 (*t*(75) = 8.30, *p* < .001, *d* = .95), and significantly more false alarms in run 3 than run 1 (*t*(75) = 6.16, *p* < .001, *d* = .71). There was a small but significant decrease in false alarms between runs 2 and 3 (*t*(75) = 2.30, *p* = .024, *d* = .26)^2^.

To establish the presence of a direct forgetting effect, the DF task was initially analysed in a 2 × 2 × 2 repeated measures ANOVA, assessing the main and interaction effects of word list (1 or 2), condition (*forget* or *remember*), and testing order (whether the *forget* condition ran at the start or end of the testing sessions). The only significant effect this produced was a condition × order interaction, *F*(1, 74) = 6.94, *p* = .010, μ^2^_p_ = .09, suggesting that testing order had affected how some participants responded to the *remember* versus *forget* cues (all other *F* < 2.80, *p* > .100). As the means in Table 1 indicate, participants in either testing order appeared to show a “cost” effect of directed forgetting (list 1 recall < list 2 recall for the *forget* condition), but only those who attempted the *remember* condition first showed the “benefit” effect (list 1 recall for *remember >* list 1 recall for forget). This was supported by a follow-up ANOVA comparing list 1 and list 2 recall for the forget condition only, including testing order as a between-subjects variable: a main effect of list was observed, *F*(1, 74) = 4.61, *p* = .035, μ^2^_p_ = .06, but neither a main effect of order nor an interaction effect were observed (all *F* < 3.20, *p* > .080). As an individual differences index of the DF effect, we therefore used the “cost” effect: list 2 recall - list 1 recall in the *forget* condition. This is equivalent to the index used by Soriano et al. (2009). No significant difference in LSHS-A scores was evident between participants who completed the different task orders (Mann–Whitney *U* = 605.00, *p* = .223, *N* = 76).

### 3.2 Relations between inhibition, directed forgetting, and hallucination-proneness

Table 2 displays the correlation matrix between performance on the ICIM, DF, source memory and hallucination-proneness. Consistent with our first hypothesis, ICIM performance positively correlated with hallucination-proneness (*p* = .046). Poorer source memory performance correlated with false alarms on the ICIM (*p* = .023) and DF performance (*p* = .034) but not hallucination-proneness (*p* = .831). DF and ICIM scores did not correlate (*p* = .785).

**Table 2:** Correlations among ICIM, signal detection, source memory, and LSHS-A scores.

	Directed Forgetting	Source Memory	LSHS-A
ICIM False Alarms (2–3)	−.03	−.26^a^	.23^a^
Directed Forgetting	–	−.24^a^	−.03
Source Memory	–	–	−.02
LSHS-A	–	–	–

a *p* < .05, two-tailed. *N* = 76.

A linear regression analysis was run using ICIM performance in block 1, DF performance in block 2, and source memory performance in block 3, with LSHS-A as the dependent variable (using log-transformed scores for ICIM and LSHS-A data). As shown in Table 3, the initial model was significant, with ICIM false alarms significantly predicting LSHS-A scores (stan. β = .243, *p* = .035), in line with hypothesis 1. However, neither the addition of DF scores in block 2 (Δ *R2* < .001, *F*(1, 73) = .04, *p* = .850) nor source memory scores in block 3 (Δ *R2* < .001, *F*(1, 73) = .03, *p* = .853) made significant contributions to the model. In the final model only ICIM scores predicted hallucination-proneness (see Table 3i.). Therefore, hypotheses 2 and 3 were not supported: any intentional inhibition effects indexed by the DF did not predict hallucination-proneness, and source memory could not account for the predictive relation evident for ICIM.

**Table 3:** Hierarchical regression analysis for i) predicting auditory hallucination-proneness and ii) intrusive thoughts.

	*B*	*SE B*	*Beta*	*t*	*p*	*C.I.(95%)*		*F*	*df*	*p*	*R*^*2*^
**i) LSHS-A**											
ICIM False Alarms	.09	.04	.24	2.15	.04	.01	.17	4.64	1, 74	.04	.06

ICIM False Alarms	.09	.04	.25	2.14	.04	.02	.48	2.31	2,73	.11	.06
Directed Forgetting	.00	.01	.02	.02	.85	−.21	.25				

ICIM False Alarms	.09	.04	.25	2.12	.04	.01	.49	1.53	3,72	.21	.06
Directed Forgetting	.00	.01	.03	.23	.82	−.21	.27				
Source Memory (Self-Other)	.09	.51	.02	.19	.85	−.22	.26				

**ii) WBSI-I**											
ICIM False Alarms	1.45	.53	.30	2.75	.01	.40	2.50	7.57	1, 74	.01	.09

ICIM False Alarms	1.37	.53	.29	2.57	.01	.06	.51	4.32	2,73	.02	.11
Directed Forgetting	−.15	.15	−.12	−1.03	.31	−.34	.11				

ICIM False Alarms	1.43	.55	.30	2.60	.01	.07	.53	2.93	3,72	.04	.11
Directed Forgetting	−.13	.15	−.10	−.86	.39	−.33	.13				
Source Memory (Self-Other)	3.20	6.53	.06	.49	.63	−.18	.29				

*N* = 76. ICIM = Inhibition of Current Irrelevant Memories.

LSHS-A = Launay-Slade Hallucination Scale – Auditory subscale. WBSI-I = White Bear Suppression Inventory – Intrusions subscale.

A number of different outcomes and approaches are favoured when indexing source memory: for example, it has been suggested that self-other errors are of particular theoretical interest (given their relevance to externalising biases), while new items on the task may also be considered “lures” to create false alarms. To examine this, we reran the final combined models of the above analyses (i.e., block 3), each time replacing the source memory score with i) self-other errors, ii) other-self errors, and iii) all new-old errors (i.e., new trials marked as “self” and new trials marked as “other”). However, this made little difference to the main results: in each case, source memory failed to predict LSHS-A score (all *p* > .05, all stan. Β < .22), while ICIM false alarms were a significant predictor in every model (.02 < *p* < .035).

To test for specificity, we also ran the above analysis with visual hallucination-proneness scores (LSHS-V) as the dependent variable (see Supplementary Materials, Table 1). Visual scores correlated with false alarms on the ICIM (*r* = .26, *p* = .02) but none of the other main task outcomes (all *r* < .11, all *p* > .37). Regression analysis using transformed ICIM and LSHS-V scores highlighted a significant relationship between the two in each block of the model, as was the case for auditory scores. However, the model did not produce normal residuals (a required assumption for regression analysis), even with the use of log-transformed variables.

### 3.3 The role of intrusive thoughts

Our fourth hypothesis was that cognitive task performance may be associated with a susceptibility to intrusive thoughts, rather than hallucination-proneness *per se*. To examine this, we first reran the above analyses with WBSI Intrusion scores as the dependent variable (with ICIM scores in block 1, DF scores in block 2, and source memory scores in block 3). This produced very similar results to those found for the LSHS-A: in the final model, only false alarms on the ICIM predicted intrusions (stan. β = .300, *p* = .011, see table 3ii), whereas no significant contributions were evident for DF and source memory (all *p* > .30). This supported hypothesis 4: ICIM performance – but not source memory or DF – was related to intrusive thoughts.

To further explore the relation between intrusions, hallucination-proneness, and ICIM performance, we then compared regression models where i) ICIM and WBSI scores predicted LSHS-A, and ii) ICIM and LSHS-A scores predicted WBSI scores. For i) predicting LSHS-A scores, the overall model was significant (*R*^*2*^ = .207, *F*(2, 73) = 9.52, *p* < .001), but when the contribution of WBSI scores was taken into account (stan. β = .404, *p* < .001, 95% C.I. = .19–.062), ICIM scores no longer predicted hallucination-proneness (stan. β = .120, *p* = .227, 95% C.I. = −.10 – .34). This was also true for the second analysis (ii), although while LSHS-A scores predicted intrusion scores (stan. β = .389, *p* < .001, 95% C.I. = .18–.60), there was only a trend for ICIM scores to still predict WBSI scores (stan. β = .210, *p* = .050, 95% C.I. = .00–.42; *R*^*2*^ = .236, *F*(2, 73) = 11.24, *p* < .001).

This suggested that intrusion scores may mediate the relation between hallucination-proneness scores and ICIM performance (i.e., hallucination-proneness acting as a mediator). To further test this, we conducted a mediation analysis using hallucination-proneness (LSHS-A score) as the dependent variable, ICIM performance (number of false alarms) as the predictor variable, and intrusive thoughts (WBSI scores) as the mediator. Mediation analysis was carried out with jamovi software, using the ‘medmod’ package (Selker, 2017). The direct effect of ICIM performance on hallucination-proneness was not significant (*B* = .04, *SE* = .04, *p* = .264). However, there was an indirect effect of ICIM performance on hallucination-proneness through intrusive thoughts (*B* = .04, *SE* = .02, *p* = .025), indicating that intrusions fully mediated the association between inhibition and hallucination-proneness.^3^

### 3.4 Accounting for relative performance: the temporal context confusion (TCC) score

One difficulty with using measures such as false alarms on the ICIM is that they do not measure errors in the context of overall task performance, whether in terms of correct responses to targets (hits) or general memory performance (e.g., run 1 scores). Since its first application in hallucinations research (Waters et al., 2003), studies using versions of the ICIM have also included a “temporal context confusion score”, which is thought to act as a marker of how much participants confuse information between ICIM lists (Nahum, Bouzerda-Wahlen, Guggisberg, Ptak, & Schnider, 2012). The TCC score is calculated by:

$$\frac{Run2FAs+Run3FAs}{Run2Hits+Run3Hits}-\frac{Run1FAs}{Run1Hits}$$

The TCC has a strong positive correlation with failures to correctly monitor other contextual information in a task (Nahum et al., 2012, Schnider et al., 1996). It also provides an index of how many mistakes participants are making relative to their own baseline of memory performance, given that errors on run 1 are thought to reflect working memory rather than inhibitory skills.

When the above analyses were rerun (see Supplementary Materials Table 2), a stronger contribution was evident for TCC score when predicting hallucination-proneness than was previously observed for false alarms (stan. β = .306, *p* = .007, 95% *C.I.* = .15–.93) and this was still the case when DF and SMT scores were added for blocks 2 and 3 (stan. β = .323, *p* = .007, 95% *C.I.* = .09–.56). For intrusive thoughts, the TCC was also a significant predictor in all three blocks, but generally with lower parameter estimates than for predicting hallucination-proneness (stan. β = .23–.25). Taken together, TCC scores from the ICIM appeared to be a potentially more sensitive index of hallucination-proneness, while false alarms were more consistently related to intrusive thoughts.

## 4 Discussion

The primary aim of this study was to test whether intentional inhibition relates to hallucination-proneness in a non-clinical sample, even when other important factors – such as source monitoring and intrusive thoughts – are taken into account. Our first hypothesis was that scores for auditory hallucination-proneness would be predicted by performance on the Inhibition of Current Irrelevant Memories (ICIM) task (hypothesis 1): this was supported, providing a replication of the effect observed by Paulik et al. (2007). This relationship with hallucination-proneness did not extend to a second test of intentional inhibition – the Directed Forgetting (DF) task (hypothesis 2) – but it was robust to the contribution of source monitoring, as indexed by a source memory task (hypothesis 3). The relationship with intrusive thoughts was more complex: false alarms on the ICIM appear to be a stronger index of intrusions than hallucination-proneness (hypothesis 4), and further analysis suggested that intrusive thoughts mediated the association between intentional inhibition and hallucination-proneness.

The significant relationship between hallucination-proneness scores and performance on the ICIM supports prior findings by Paulik et al. (2007), who found group differences on ICIM performance between those high and low in hallucination-proneness (see also Badcock et al., 2015). Our study builds on this by demonstrating that this is unlikely to result from source-monitoring demands on the ICIM. Instead, it supports the idea that intentional cognitive inhibition plays an important part in susceptibility to hallucinatory experiences (Badcock et al., 2005, Waters et al., 2003) and may show potential as a transdiagnostic marker of AH (Badcock & Hugdahl, 2014).

However, the lack of any relation between DF performance and hallucination-proneness suggests that the concept of intentional inhibition requires further close examination. If it is a viable cognitive marker (of hallucinations or intrusions), then other inhibition paradigms should also be able to demonstrate links with psychopathology-like traits in the same way as the ICIM; both paradigms have been used as specific examples of intentional and conscious suppression of information from working memory, as opposed to more automatic and association-based intrusions into recall (Paulik et al., 2008, Racsmány et al., 2008). In our data, one possibility is that the presence of order effects between participants on the DF task may have obscured a more robust forgetting effect, in that participants who completed the *forget* condition first may not have encoded list 1 of the *remember* condition as strongly if they were expecting another forget cue to appear. The within-subjects design used here – sometimes referred to as a “four list” design (Sahakyan et al., 2013) – is not common compared to between-subjects approaches (Conway et al., 2000), but when such designs are used, they have been robust to order effects (e.g., Zellner & Bäuml, 2006). Moreover, we did observe a “cost” effect between the two *forget* lists, despite differences in condition order: this effect in particular has been previously related to hallucination severity in patients (Soriano et al., 2009). As such, it is unclear whether establishing a more robust DF effect would have substantively changed the main results regarding hallucination-proneness.

Another possibility is that the DF paradigm does not actually index inhibition skills: it has been argued that context changes between word lists drive the effect instead (Sahakyan et al., 2013) and the role of inhibition in similar paradigms – such as Think/No Think tasks – has been questioned (Noreen & MacLeod, 2015). However, the wide range of groups who struggle with such tasks include many who are susceptible to problems with inhibition, including older adults (Aslan & Bäuml, 2013) and people with Alzheimer's Disease (Haj, Fasotti, & Allain, 2014), Obsessive Compulsive Disorder (Konishi, Shishikura, Nakaaki, Komatsu, & Mimura, 2011), and Post-Traumatic Stress Disorder (Cottencin et al., 2006). Further examination of intentional inhibition in relation to alternative measures of cognitive inhibition are required to establish why only certain kinds of memory suppression are related to psychopathological traits.

A key finding from our dataset that goes beyond merely noting associations between inhibition and hallucination-proneness concerns intrusion: our data seem to suggest that tasks like the ICIM pick out a general tendency to experience intrusive cognitions, and this may mediate the path to hallucination-like experiences. This is consistent with long-standing ideas that auditory hallucinations occur when intrusive cognitions are attributed to a non-self source because they are ego-dystonic in some way (i.e., unacceptable to one's conception of self; Morrison et al., 1995). Beyond their relevance to the intrusive content of AH, problems with managing unwanted thoughts and impulses are potentially relevant to both the role of top-down expectation in perception (Powers, Kelley, & Corlett, 2016) and the understanding of how executive control difficulties potentially impact upon the management of unusual and distressing experiences (Hugdahl, 2009). Inhibitory skills are also affected by sleep problems (Petrovsky et al., 2014), and hallucinatory experiences around the boundaries of sleep are known to be common (Jones et al., 2009, Reeve et al., 2015).

Aside from the inhibition-related results, the lack of evidence for a source memory effect has important implications in itself. Following Garrison et al. (2017) this provides another example of how source monitoring in general, and source memory in particular, may not be part of a cognitive “continuum” for hallucinations and hallucination-like experiences (cf. Brookwell et al., 2013). Indeed, problems with monitoring distinctions of self–other and reality–fantasy may be a key dividing line for those with frequent hallucinatory experiences who do or do not present to mental health services. The existence of a continuum of such experiences is sometimes talked about as if it is an “either/or” question (David, 2010, Lawrie et al., 2010, Stanghellini et al., 2012). In contrast, we would argue that the phenomenological features of hallucinatory states are likely to reflect independent underlying cognitive processes, which will vary in how continuously they are distributed in the wider, non-clinical population. Source memory – picking out a more fundamental disorientation of self and other – may not show such continuity between clinical and non-clinical groups, while intentional inhibition – tracking intrusive and uncontrollable cognitive states – may do so. When one considers the potential additional roles of perceptual bias (Moseley, Smailes, Ellison, & Fernyhough, 2016), attentional control (Hugdahl, 2009), and agency detection (Stuke, Kress, Weilnhammer, Sterzer, & Schmack, 2018), it seems plausible that any phenomenological continuum of hallucinations is in fact highly likely to be underpinned by at least some discontinuities at the cognitive and neural levels.

Tracking such continuities and discontinuities requires careful analysis of the different tasks and processes implicated in hallucination-proneness to date. Given the prevalence of small effects and heterogenous methods in “analogue” studies, a key aim must be replicability: with this in mind, we are currently part of an international consortium which is testing many of the “classic” cognitive tasks linked to hallucination-proneness in a sample of over 800 healthy individuals (Moseley, 2018). It will also be important to deploy similar tasks with people who have frequent (i.e., daily or weekly) hallucinatory experiences, such as non-clinical voice-hearers (Alderson-Day et al., 2017, Sommer et al., 2010). Research with such cohorts has included some cognitive assessments (e.g., Daalman, Verkooijen, Derks, Aleman, & Sommer, 2012) but has not always included standard measures of source memory or auditory signal detection.

A further observation from our data regarded associations between ICIM performance and proneness to visual hallucinations. Using scores from the visual subscale of the LSHS in the regression analysis indicated a similar association between intentional inhibition and visual hallucinations (albeit while producing a non-normal distribution of residuals). This might imply that intentional inhibition not only underlies auditory hallucinations, but also those in the visual modality. Further research is needed to explore links between inhibition and hallucinations in specific modalities (for an example of this approach, see Aynsworth et al., 2017, Smailes et al., 2018); the data presented here are merely suggestive of inhibition as a process relevant to hallucinations across a range of modalities. The results for the temporal context confusion score (and its apparently stronger relation to hallucination-proneness than intrusive thoughts) also highlight the need for taking into account contextual factors on the ICIM: we recommend that future studies using the task deploy the TCC as their primary outcome for individual differences analysis of hallucinatory traits, which would also align with the more recent use of similar continuous recognition tasks (e.g., Wahlen, Nahum, Gabriel, & Schnider, 2011).

These findings have some limitations which need to be considered when interpreting the results. First, we were reliant on participants’ self-reported proneness to hallucinations and intrusions, and did not assess them in person regarding their mental health or other potential confounds, such as substance use history. Necessarily, this limits what can be said about the sample tested: on the one hand, some of the sample may have previously received psychiatric diagnoses and may have more in common with a clinical cohort; on the other hand, levels of overall hallucination-proneness may have been too low to effectively pursue our research questions. While the former is arguably unlikely for a young, university-based sample, it is possible that deploying a more extensive pre-screening stage, or preselecting high and low hallucination-proneness groups, would have increased the range of our questionnaire data. However, as shown in Fig. 1, a substantial minority of our sample scored at 50% or higher on the LSHS-A, requiring them to endorse “Often” or “Almost Always” for a number of hallucination items. Moreover, the pattern of results observed here (replication of a relation to ICIM performance and lack of a source memory effect) would not easily be explained by levels of hallucination-proneness being too low in the sample overall.

A second concern regards gender. Gender imbalances are common in university samples (Dickinson, Adelson, & Owen, 2012) and the large number of female compared to male participants in the present study precludes generalisations to the general population. It is possible that the use of a male voice on the source memory task in particular may have affected performance in a largely female cohort. We have, however, previously observed a similar null effect (i.e., no relation between source memory performance and hallucination-proneness) on a task with a female speaker and similar gender ratio (Garrison et al., 2017). Finally, without a larger battery of classic inhibition tasks (such as the Stroop or Flanker paradigms), these data cannot show that intentional cognitive inhibition is the only kind of inhibitory control relevant to hallucinations and intrusions. Prior studies have demonstrated this specificity (e.g., Paulik et al., 2008), but a more extensive analysis of task demands and executive functioning components of intentional inhibition – perhaps via latent variable modelling (Friedman and Miyake, 2004, Miyake et al., 2000) – would be an important avenue for future research. Other paradigms that appear to track memory intrusions will also be important to include in this endeavour (Brébion et al., 2009, Sugimori et al., 2011).

## 5 Conclusion

In conclusion, intentional inhibition is a cognitive mechanism that can be related to hallucination-proneness and intrusive thoughts. This appears to be largely independent of source monitoring ability and may act as a marker of a potential cognitive continuum underlying proneness to unusual experiences - at least for non-clinical populations. Further examination of inhibitory skills in people with frequent hallucinations is required to understand more about how the merely intrusive becomes the uncontrollable, spontaneous, and perceptual.

## Footnotes

1 Healthy participants were included in Soriano et al. (2009) but only as control participants, without a measure of their own potential proneness to hallucinations.

2 As can be seen in Fig. 2, there were a number of high-scoring participants for false alarms on runs 2 and 3 that could be considered outlier cases. As the removal of these cases made little difference to the results of the repeated measures ANOVA or subsequent regression analysis, they were included for the remainder of the analyses; any departures from the main results are noted below.

3 As before, this analysis was repeated after excluding the five participants who made very high number of false alarms in runs 2 or 3. In this case, after exclusion, the indirect effect of ICIM false alarms on hallucination-proneness through intrusive thoughts became non-significant (*B* = .40*, SE* = .02, *p =* .052).
