# Supplementary material for: Intentional inhibition but not source memory is related to hallucination-proneness and intrusive thoughts in a university sample
Source: Cortex. 2019 Apr;113:267–78. doi: 10.1016/j.cortex.2018.12.020 (PMC6459394; doi:10.1016/j.cortex.2018.12.020)
Supplement: Multimedia component 1 [file mmc1.docx]

**Supplementary Table 1. Hierarchical regression analysis for predicting visual hallucination-proneness using the ICIM, directed forgetting and source memory tasks**

|  | *B* | *SE B* | *Beta* | *t* | *p* | *C.I.(95%)* | | *F* | *df* | *p* | *R2* |
| --- | --- | --- | --- | --- | --- | --- | --- | --- | --- | --- | --- |
| **i) LSHS-V** |  |  |  |  |  |  |  |  |  |  |  |
| ICIM False Alarms | 0.09 | 0.04 | 0.24 | 2.10 | 0.04 | 0.01 | 0.17 | 4.42 | 1, 74 | 0.04 | 0.06 |
|  |  |  |  |  |  |  |  |  |  |  |  |
| ICIM False Alarms | 0.09 | 0.04 | 0.24 | 2.09 | 0.04 | 0.00 | 0.17 | 2.19 | 2,73 | 0.12 | 0.06 |
| Directed Forgetting | 0.00 | 0.01 | 0.02 | 0.15 | 0.88 | -0.02 | 0.03 |  |  |  |  |
|  |  |  |  |  |  |  |  |  |  |  |  |
| ICIM False Alarms | 0.10 | 0.04 | 0.26 | 2.21 | 0.03 | 0.03 | 0.50 | 1.67 | 3,72 | 0.18 | 0.06 |
| Directed Forgetting | 0.00 | 0.01 | 0.04 | 0.35 | 0.72 | -0.20 | 0.28 |  |  |  |  |
| Source Memory (Self-Other) | 0.41 | 0.52 | 0.10 | 0.80 | 0.43 | -0.14 | 0.34 |  |  |  |  |

*N* = 76. ICIM = Inhibition of Current Irrelevant Memories. LSHS-V = Launay-Slade Hallucination Scale – Visual subscale. WBSI-I = White Bear Suppression Inventory – Intrusions subscale.

**Supplementary Table 2. Hierarchical regression analysis for i) predicting auditory hallucination-proneness and ii) intrusive thoughts using the temporal context confusion (TCC) score**

|  | *B* | *SE B* | *Beta* | *t* | *p* | *C.I.(95%)* | | *F* | *df* | *p* | *R2* |
| --- | --- | --- | --- | --- | --- | --- | --- | --- | --- | --- | --- |
| **i) LSHS-A** |  |  |  |  |  |  |  |  |  |  |  |
| ICIM TCC | 0.54 | 0.20 | 0.31 | 2.77 | 0.01 | 0.15 | 0.93 | 7.66 | 1, 74 | 0.01 | 0.09 |
|  |  |  |  |  |  |  |  |  |  |  |  |
| ICIM TCC | 0.54 | 0.20 | 0.31 | 2.75 | 0.01 | 0.08 | 0.53 | 3.78 | 2,73 | 0.03 | 0.09 |
| Directed Forgetting | 0.00 | 0.01 | -0.01 | -0.05 | 0.96 | -0.23 | 0.22 |  |  |  |  |
|  |  |  |  |  |  |  |  |  |  |  |  |
| ICIM TCC | 0.57 | 0.21 | 0.33 | 2.82 | 0.01 | 0.09 | 0.56 | 2.58 | 3,72 | 0.060 | 0.10 |
| Directed Forgetting | 0.00 | 0.01 | 0.01 | -0.09 | 0.93 | -0.22 | 0.24 |  |  |  |  |
| Source Memory (Self-Other) | 0.25 | 0.51 | 0.08 | 0.69 | 0.49 | -0.18 | 0.30 |  |  |  |  |
| **ii) WBSI-I** |  |  |  |  |  |  |  |  |  |  |  |
| ICIM TCC | 5.45 | 2.63 | 0.23 | 2.07 | 0.04 | 0.20 | 10.69 | 4.29 | 1, 74 | 0.04 | 0.06 |
|  |  |  |  |  |  |  |  |  |  |  |  |
| ICIM TCC | 5.33 | 2.62 | 0.23 | 2.04 | 0.05 | 0.00 | 0.45 | 3.07 | 2,73 | 0.05 | 0.08 |
| Directed Forgetting | -0.20 | 0.15 | -0.15 | -1.34 | 0.18 | -0.38 | 0.07 |  |  |  |  |
|  |  |  |  |  |  |  |  |  |  |  |  |
| ICIM TCC | 5.72 | 2.75 | 0.22 | 2.08 | 0.04 | 0.01 | 0.48 | 2.11 | 3,72 | 0.11 | 0.08 |
| Directed Forgetting | -0.18 | 0.15 | -0.15 | -1.17 | 0.25 | -0.37 | 0.10 |  |  |  |  |
| Source Memory (Self-Other) | 3.39 | 6.74 | -0.02 | 0.50 | 0.62 | -0.18 | 0.30 |  |  |  |  |

*N* = 76. ICIM = Inhibition of Current Irrelevant Memories. LSHS-A = Launay-Slade Hallucination Scale – Auditory subscale. WBSI-I = White Bear Suppression Inventory – Intrusions subscale.
